# ONEST: a web-based platform for the rapid and robust analysis of protein excited states through CEST spectroscopy

**DOI:** 10.1093/bioinformatics/btag158

**Published:** 2026-03-27

**Authors:** Joonhyeok Choi, Sang-Yeop Lee, Kyungdoe Han, Marta G Carneiro, Kyoung-Seok Ryu, Donghan Lee

**Affiliations:** Center for Protein Structure and Drug Mechanism Research, Korea Basic Science Institute, Cheongju, 28119, Republic of Korea; Department of Applied Pharmacy, College of Pharmacy, Chungbuk National University, Cheongju, 28160, Republic of Korea; Center for Target-to-Therapeutics Research, Korea Basic Science Institute, Cheongju, 28119, Republic of Korea; Department of Convergent Analytical Science, University of Science and Technology, Daejeon, 34113, Republic of Korea; Department of Civil and Environmental Engineering, University of Wisconsin—Madison, Madison, WI 53706, United States; ZoBio B.V, Leiden, 2333 CH, The Netherlands; Center for Protein Structure and Drug Mechanism Research, Korea Basic Science Institute, Cheongju, 28119, Republic of Korea; Department of Convergent Analytical Science, University of Science and Technology, Daejeon, 34113, Republic of Korea; Center for Protein Structure and Drug Mechanism Research, Korea Basic Science Institute, Cheongju, 28119, Republic of Korea; Department of Convergent Analytical Science, University of Science and Technology, Daejeon, 34113, Republic of Korea

## Abstract

**Summary:**

The biological function of proteins is often driven by “invisible” excited states—transient, low-population conformations that remain undetectable by conventional structural methods. Chemical Exchange Saturation Transfer (CEST) NMR spectroscopy is a powerful technique for characterizing these states; however, the complexity of data analysis and the computational cost of numerical fitting have hindered its widespread adoption. To address these challenges, we present ONEST (Optimized Novel Exchange Saturation Transfer), a user-friendly web server designed to automate and accelerate CEST analysis. ONEST utilizes a simultaneous multi-field fitting algorithm that leverages exact analytical solutions for two-state exchange (Baldwin model) rather than computationally intensive numerical integration. This approach incorporates a rigorous correction for radio-frequency (RF) field inhomogeneity and resolves parameter degeneracy by jointly fitting datasets acquired at distinct RF field strengths. Validation against synthetic datasets yielded reduced c2 values near unity (∼1.05), confirming that the analytical approach recovers kinetic parameters with accuracy comparable to full Bloch-McConnell simulations but at a fraction of the computational cost. Furthermore, application to the anti-HIV lectin OAA successfully characterized slow conformational exchange (k_ex_ = 279 s^−1^) involving a minor population of 3%. By streamlining the extraction of kinetic and thermodynamic parameters, ONEST significantly lowers the technical barrier to entry, enabling a broader range of researchers to investigate protein dynamics at atomic resolution.

**Contact:**

dlee04@kbsi.re.kr

**Availability and implementation:**

ONEST is available through the web server at http://onest.ai.kr.

## 1 Introduction

Protein dynamics spanning many orders of magnitude in time are essential for biological processes (Palmer 2004, Ban et al. 2012, Ban et al. 2013). In particular, slow time-scale (slower than several hundreds of microseconds) global motions, such as conformational interconversions (Carneiro et al. 2015a) and domain rearrangements (Tzeng and Kalodimos 2012), are often mediated by “excited” or “invisible” states. These invisible states are transient, sparsely populated conformations (Vallurupalli et al. 2008, Vallurupalli et al. 2012) that exist at extremely low populations (<5%) (Baldwin and Kay 2009). Although these states comprise only a few percent of the population, those can be functionally important, often associated with transition conformations and/or on-pathway intermediates in biological processes (Korzhnev and Kay 2008, Baldwin and Kay 2009).

While X-ray crystallography and cryo-EM excel at visualizing the most stable, major conformational states of biomolecules, they often miss the transient, “invisible” states that drive function. Nuclear Magnetic Resonance (NMR) spectroscopy fills this important gap for understanding the function of biomolecules. Specifically, the Chemical Exchange Saturation Transfer (CEST) technique offers a unique capability to detect and characterize these functional invisible states (Bouvignies and Kay 2012, Vallurupalli et al. 2017).

The core principle of Chemical Exchange Saturation Transfer (CEST) is the application of a selective radio-frequency (RF) field to saturate the magnetization of nuclei in a sparsely populated, invisible state. Through chemical exchange, this saturation is transferred to nuclei in the ground (major) state, resulting in a measurable attenuation of the major-state NMR signal (Luz and Meiboom 1963). This mechanism effectively amplifies the signals of otherwise invisible states, making CEST a powerful tool for characterizing protein dynamics on the slow-to-intermediate timescale. However, this heightened sensitivity comes at the cost of increased experimental and computational complexity.

While recent developments in pulse-sequence design have substantially reduced acquisition times (Cabrera Allpas et al. 2022), data analysis remains a primary bottleneck. A major challenge is the strong interdependence among kinetic and thermodynamic parameters, specifically the exchange rate (k_ex_), the excited-state population (p_B_), and the minor-state transverse relaxation rate (R_2,B_). These parameters cannot be uniquely determined from data acquired at a single RF field strength. Furthermore, conventional fitting methods rely on the numerical integration of the Bloch–McConnell equations; while accurate, this approach is computationally expensive (Carneiro et al. 2015b).

To alleviate these issues, simplified analytical models for two-state exchange—particularly those utilizing analytical expressions for the R_1ρ_ relaxation rate—have gained traction (Miloushev and Palmer 2005, Baldwin and Kay 2013). These strategies offer accuracy nearly identical to full numerical solutions (Bloch 1946, McConnell 1958) while vastly improving computational efficiency (Jiang et al. 2016). The rationale for the two-state approximation is well-supported in recent methodological reviews (e.g., (Anthis and Clore 2015, Bhattacharyya et al. 2022)). Although biomolecular dynamics play out over complex energy landscapes, the effective exchange detected at a specific nucleus is frequently dominated by a single major kinetic pathway. Under such conditions, a two-state model captures the essential physics without the over-parameterization that leads to ill-posed fits and excessive computational cost. This simplification enhances robustness, reduces parameter degeneracy, and enables the extraction of meaningful structural information.

Notably, analytical multi-profile fitting, such as the simultaneous analysis of datasets acquired at distinct RF field strengths, can further break parameter degeneracy, allowing for the reliable determination of rates and populations without resorting to heavy numerical solvers. Despite these advantages, most existing implementations require specialized programming skills, hindering broader adoption among general life-science researchers (Mangul et al. 2019).

To overcome these barriers, we introduce web-based Optimized Novel Exchange Saturation Transfer (ONEST) platform, a user-friendly software tool designed to extract structural and kinetic information on invisible excited states. By leveraging the practical and theoretical benefits of analytical two-state modeling, ONEST makes advanced CEST analysis accessible without requiring expert knowledge in numerical simulation or custom coding.

## 2 Implementation

### 2.1 Software workflow and input data

**Figure 1:**
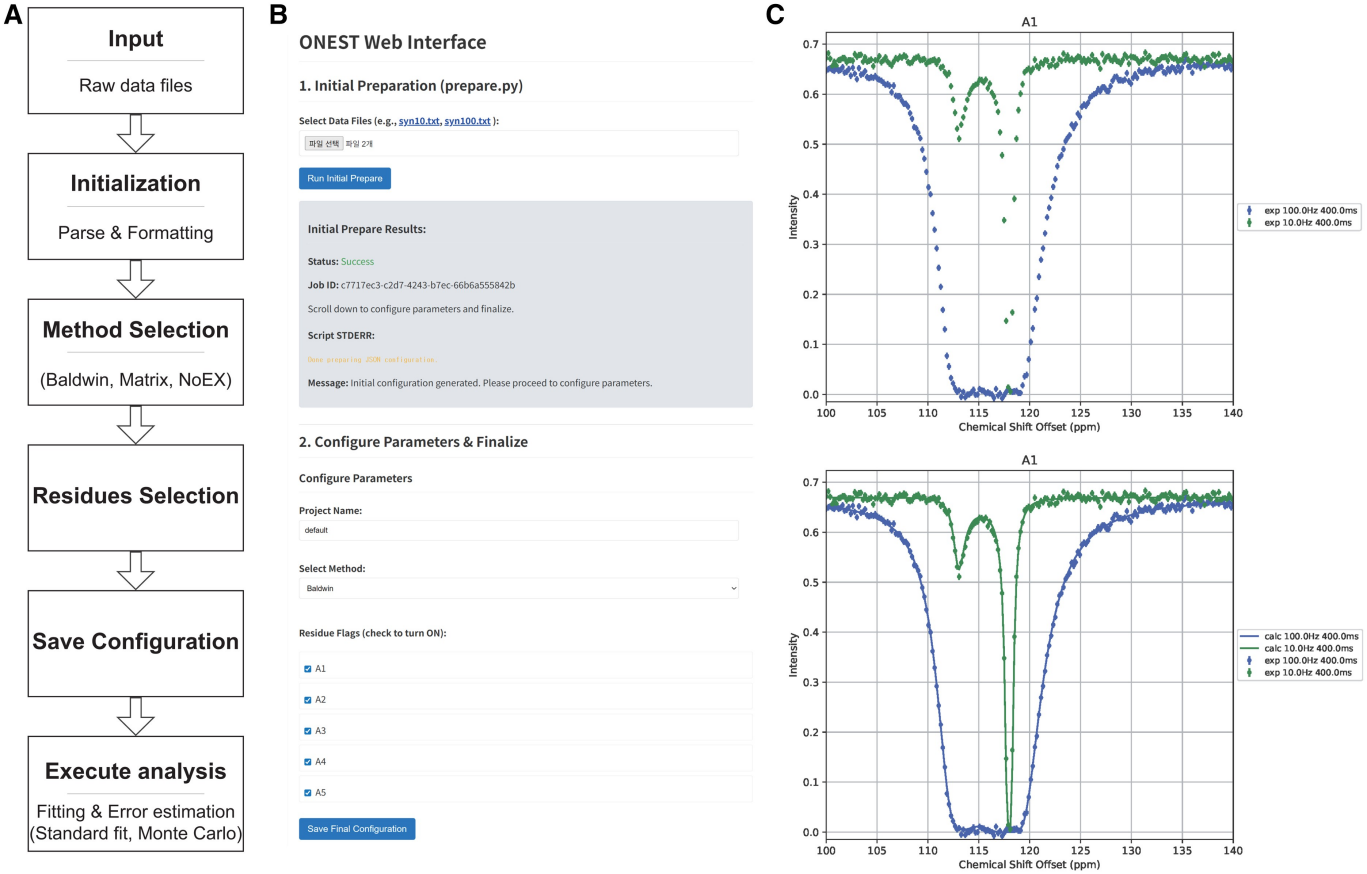
Overview of the ONEST software workflow, interface, and output. (A) Schematic flowchart illustrating the stepwise analysis pipeline. The process is structured into six sequential modules: (1) Input, where raw data is provided; (2) Initialization, which involves parsing and formatting the uploaded data; (3) Method Selection, allowing users to choose between the Baldwin analytical, Matrix numerical, or NoEx baseline models; (4) Residue Selection, for filtering specific peaks of interest; (5) Configuration Saving, which ensures reproducibility by archiving project settings; and (6) Execution, where final parameter optimization is performed using standard least-squares fitting or Monte Carlo simulations for robust error estimation. (B) The web-based user interface corresponding to the setup phases. The screenshot highlights the “Initial Preparation” section for uploading plain-text data files and the “Configure Parameters” section for selecting the analysis method. (C) Representative analysis results for residue A1. The upper panel displays the input experimental CEST profiles acquired at two distinct radio-frequency (RF) field strengths (10 Hz, blue diamonds; 100 Hz, green diamonds). The lower panel shows the experimental data overlaid with theoretical fit curves (solid lines) calculated using the Baldwin method.

The ONEST pipeline is designed to provide an intuitive and efficient framework for analyzing CEST data (Fig. 1A). The workflow begins with Input Initialization, where users upload experimental datasets as plain-text tables. To overcome intrinsic parameter degeneracy in CEST analysis, the software requires two datasets acquired at different RF field strengths. Each file must follow a standard column-based format, chemical shift offset versus normalized signal intensity, consistent with established conventions in NMR relaxation-dispersion experiments.

Following data ingestion, the workflow proceeds through parameter specification and residue identification before the final numerical fitting. Users configure global project parameters—such as the static magnetic field (*B*_0_), saturation pulse duration (T_sat_), and specific RF field strengths (*B*_1_)—which serve as essential constants for the optimization algorithm. Subsequently, residues of interest are selected through a streamlined web interface (Fig. 1B). In this step, users can filter the dataset based on signal-to-noise thresholds or manually identify residues exhibiting characteristic exchange-induced dips, ensuring that the analysis remains focused on the most relevant spectral regions for robust parameter extraction.

### 2.2 Mathematical modeling and analysis kernels

To accommodate diverse computational requirements, ONEST provides three complementary analysis engines, each corresponding to a distinct modeling strategy.

1. No-Exchange Baseline (NoEx): This module serves as a negative control, computing the expected signal intensities under the assumption that no chemical exchange occurs. Functionally, it performs a simplified on-resonance *B*_1,_ calculation, enabling users to assess baseline spectral quality and verify reference relaxation parameters prior to exchange modeling.
2. Numerical Integration (Matrix Method): For high-fidelity simulations, ONEST implements a full numerical treatment of two-state chemical exchange. The software constructs the complete 7 × 7 Bloch–McConnell evolution matrix and evaluates magnetization propagation via matrix exponentiation. Although computationally demanding, this approach yields an exact numerical solution and provides a benchmark for validating analytical approximations.
3. Analytical Approximation (Baldwin Method): To support high-throughput fitting, ONEST incorporates the exact analytical solution developed by (Baldwin 2014). This method calculates the effective rotating-frame relaxation rate *R*_1__ρ_ and predicts the corresponding signal intensity *I*_calc_. It achieves accuracy that closely matches the Matrix method while dramatically reducing computational cost, making it the preferred option for large-scale datasets or iterative parameter optimization.

### 2.3 Correction for RF field inhomogeneity

To enhance robustness under realistic experimental conditions, ONEST incorporates a correction for spatial variation in the spin-lock field (*B*_1_). The software models RF inhomogeneity as a Gaussian distribution across the sample volume. During analysis, calculated signal intensities are averaged and weighted according to this distribution, compensating for deviations in *B*_1_ that might otherwise introduce systematic bias into the extracted exchange parameters.

## 3 Results

### 3.1 Validation on synthetic datasets

To assess the accuracy and reliability of the ONEST workflow (Fig. 1A), we generated a synthetic dataset simulating CEST measurements for five test residues (A1–A5) with the complete Bloch–McConnell evolution matrix. These residues were assigned chemical shifts of ground states (118.0, 110.0, 115.0, 120.0, and 125.0 ppm) and their chemical shift differences between the ground and excited states (Δδ = −5.0, −1.0, 2.0, 2.0, and −3.0 ppm), allowing for evaluation across multiple exchange regimes. All simulations assumed a static magnetic field corresponding to a ^15^N Larmor frequency of 80.12 MHz, a saturation time (T_sat_) of 400 ms, and kinetic parameters of forward exchange rate (k_AB_ = 15 s^−1^) and reverse exchange rate (k_BA_ = 285 s^−1^). Crucially, the test dataset included two CEST profiles per residue acquired at distinct RF field strengths (10 Hz and 100 Hz) to resolve parameter degeneracy. For accounting experimental uncertainties, the synthetic data contained 2% errors in the RF field strengths and the intensities. As shown in Figures 1B and 1C, ONEST accurately recovered the preset simulation parameters evidenced with quantitative assessment of the fit quality, reduced chi-square (*χ*^2^) values of 1.05 for the Baldwin method and 0.98 for the Matrix method (Table 1). These *χ*^2^ values, being very close to unity, statistically confirm that the software successfully identified the input parameters used to generate the simulations.

**Table 1:** Comparison of methods, fitted parameters, and computational time.

Method	k_AB_	k_BA_	Reduced χ^2^	Processing Time
Baldwin	14.4 ± 0.1	290.7 ± 2.8	1.05	3.4 sec ± 0.1
Matrix	14.9 ± 0.1	283.6 ± 2.8	0.98	28.6 sec ± 0.3
NoEX	–	–	54.9	2.7 sec ± 0.1

### 3.2 Performance on experimental biological systems

We next evaluated ONEST using experimental CEST data from the anti-HIV lectin OAA, a protein previously shown to undergo slow conformational exchange at 277 K (Carneiro et al. 2015a). The resulting model fits closely reproduced the experimental Z-spectra, capturing both the overall line shape and subtle attenuation features associated with chemical exchange. Fit quality was reflected in consistently low reduced *χ*^2^ values, confirming that ONEST effectively handles realistic spectral noise, heterogeneous baselines, and complex relaxation behavior. These results validate the utility of the workflow for challenging biological systems.

### 3.3 Optimization algorithm efficiency

The numerical stability observed in these analyses arises from the software’s two-stage optimization framework. Upon activation of target residues, ONEST first generates automatic initial parameter estimates, including chemical shift difference (Δδ), exchange rate (k_ex_), and minor-state population (p_B_), using the Baldwin analytical approximation. These physically grounded estimates are then refined through nonlinear least-squares optimization using the Trust-Region Reflective algorithm (SciPy; (Virtanen et al. 2020)). This strategy minimizes the risk of convergence to local minima, ensuring reliable fitting performance without the need for manual parameter tuning.

## 4 Discussion

In this study, we applied the ONEST framework to both synthetic and experimental datasets to evaluate its efficacy in quantifying slow-to-intermediate exchange processes. Analysis of the anti-HIV lectin OAA revealed that the protein exchanges between a dominant ground state (97%) and a sparsely populated excited state (3%) with a relatively slow exchange rate (k_ex_ = 279 s^−1^). Notably, the large chemical shift differences observed for residues N75 and W77 (2.28 and 6.48 ppm, respectively) underscore their specific involvement in these conformational transitions. These findings reinforce the unique capacity of CEST to detect structurally significant, low-abundance protein states that are invisible to standard techniques.

A critical feature of our workflow is the utilization of dual RF field strengths (10 Hz and 100 Hz). The weak field regime effectively constrains the chemical-shift offsets, whereas the strong field regime enhances sensitivity to relaxation and exchange contributions (Carneiro et al. 2015b) Jointly fitting these datasets significantly reduces parameter degeneracy, enabling the accurate and simultaneous determination of k_ex_, p_B_, and R_2,B_.This multi-field synergy is essential for resolving the known mathematical correlations between exchange parameters (e.g., k_ex_ vs R_2,B_) that often hamper single-field analysis. The analytical Baldwin framework demonstrated excellent performance across all tested conditions, producing fits nearly indistinguishable from full Bloch–McConnell simulations while dramatically reducing computational cost (Baldwin 2014, Jiang et al. 2016). However, limitations remain. Regimes involving very slow exchange, where saturation transfer is inherently inefficient, or multi-state models (involving >2 states) still pose challenges for simplified analytical fitting. Addressing these complex scenarios typically requires higher-dimensionality data, such as multiple B_1_ fields or B_0_ static field strengths, or complementary relaxation experiments like R_1ρ_, CPMG, or DEST (Vallurupalli et al. 2017, Palmer and Koss 2019). Future extensions of ONEST will aim to incorporate these modalities.

## 5 Conclusions

This work demonstrates that ONEST provides a fast, accurate, and user-friendly solution for characterizing low-population, slow-exchanging protein states via CEST. Rather than merely reiterating the established utility of CEST, our results show that the ONEST framework enables the reliable extraction of exchange parameters with substantially reduced computational overhead. Across both synthetic and experimental datasets, ONEST reproduced exchange profiles with high fidelity (reduced *χ*^2^ = 1.05) and achieved numerical accuracy comparable to rigorous Bloch–McConnell numerical simulations. By leveraging robust analytical solutions and multi-field fitting, the software overcomes common analysis obstacles such as parameter degeneracy and user-dependent initialization bias. Consequently, ONEST lowers the technical barrier for studying biologically critical processes dominated by “invisible” states, including amyloid fibrillation and oligomerization equilibria (Karamanos et al. 2015), making advanced CEST analysis practical for routine application.

## Data Availability

The program underlying this article is available on the project website at http://onest.ai.kr.
